# Behavioral buffering of global warming in a cold‐adapted lizard

**DOI:** 10.1002/ece3.2216

**Published:** 2016-06-12

**Authors:** Zaida Ortega, Abraham Mencía, Valentín Pérez‐Mellado

**Affiliations:** ^1^ Department of Animal BiologyUniversity of SalamancaCampus Miguel de Unamuno37007SalamancaSpain

**Keywords:** Behavioral thermoregulation, cold‐adapted, global warming, *Iberolacerta*, lizard, temperature

## Abstract

Alpine lizards living in restricted areas might be particularly sensitive to climate change. We studied thermal biology of *Iberolacerta cyreni* in high mountains of central Spain. Our results suggest that *I. cyreni* is a cold‐adapted thermal specialist and an effective thermoregulator. Among ectotherms, thermal specialists are more threatened by global warming than generalists. Alpine lizards have no chance to disperse to new suitable habitats. In addition, physiological plasticity is unlikely to keep pace with the expected rates of environmental warming. Thus, lizards might rely on their behavior in order to deal with ongoing climate warming. Plasticity of thermoregulatory behavior has been proposed to buffer the rise of environmental temperatures. Therefore, we studied the change in body and environmental temperatures, as well as their relationships, for *I. cyreni* between the 1980s and 2012. Air temperatures have increased more than 3.5°C and substrate temperatures have increased by 6°C in the habitat of *I. cyreni* over the last 25 years. However, body temperatures of lizards have increased less than 2°C in the same period, and the linear relationship between body and environmental temperatures remains similar. These results show that alpine lizards are buffering the potential impact of the increase in their environmental temperatures, most probably by means of their behavior. Body temperatures of *I. cyreni* are still cold enough to avoid any drop in fitness. Nonetheless, if warming continues, behavioral buffering might eventually become useless, as it would imply spending too much time in shelter, losing feeding, and mating opportunities. Eventually, if body temperature exceeds the thermal optimum in the near future, fitness would decrease abruptly.

## Introduction

Temperature is probably the environmental factor that most affects animals, particularly ectotherms, conditioning all aspects of their lives, survival, and, eventually, fitness (Huey and Stevenson 1979; Adolph and Porter 1993; Angilletta 2009). Environmental temperatures available for organisms vary with latitude, altitude, weather conditions, and habitat composition (Díaz et al. 2006; Deutsch et al. 2008; Sears et al. 2011; Graae et al. 2012). As it relates to temperature, animals show two dimensions: thermal sensitivity and thermoregulation (Angilletta 2009). On one hand, thermal sensitivity describes the extent to which physiological performance of an organism depends on temperature, with a gradient between thermal specialists, whose performance is optimal in a narrow range of temperatures, and thermal generalists, which are able to perform well in a wide range of temperatures (Huey and Hertz 1984; Angilletta et al. 2003; Angilletta 2009). On the other hand, thermoregulation is the ability to actively regulate body temperature, with a gradient from thermoconformers, whose body temperatures would always be similar to ambient temperatures, to perfect thermoregulators, whose body temperatures would be independent of ambient temperature (Heath 1970; Huey 1974; Hertz et al. 1993; Sears and Angilletta 2015).

There is a recent source of thermal variation affecting animals: anthropogenic climate change. The rapid increase in environmental temperatures is the main effect of this climate change (Solomon et al. 2007; Diffenbaugh and Field 2013). Global warming has a great impact on animal and plant species worldwide (Parmesan 2006; Charmantier et al. 2008; Logan et al. 2013; Kaspari et al. 2015). Among reptiles, global warming may impose an important threat of extinction (Sinervo et al. 2010; but see Huey et al. 2010). In particular, Mediterranean montane lizards of southwestern Europe would face an additional impact, due to a predicted increase in drought in this area (Araújo et al. 2006; Nogués‐Bravo et al. 2008; Carvalho et al. 2010; Ceia‐Hasse et al. 2014). It has been reported that climate warming will benefit the fitness of a thermal generalist lizard inhabiting cold areas (Chamaillé‐Jammes et al. 2006), but it would not be the case for some restricted montane cold specialists (Huey et al. 2012).

Given the global warming scenario, lizards would have two ways to avoid extinction: migration to thermal suitable areas or adaptation to new thermal conditions (Berg et al. 2010; Gunderson and Stillman 2015.). The first option is unlikely for montane lizards as they would eventually run out of space (Araújo et al. 2006; Huey et al. 2010) and most of the populations are isolated from each other with lowland barriers that preclude dispersal. The second option includes the physiological adaptation to new thermal conditions or the behavioral buffering of warming (Huey et al. 2012; Gunderson and Stillman 2015). However, thermal plasticity of physiological traits of lizards might have a limited potential, particularly the plasticity of the critical thermal maximum (Gunderson and Stillman 2015). In addition, adaptation to new conditions *in situ* would imply rates of thermal niche evolution much faster (>10.000 times) than historically experienced (Quintero and Wiens 2013). Thus, physiological adaptation would probably not be enough to keep pace with the rate of warming expected in this century. Nonetheless, lizards might buffer the negative impact of global warming by means of their thermoregulatory behavior (Huey et al. 2003; Huey and Tewksbury 2009; Kearney et al. 2009). In that way, lizards would be able to attain suitable body temperatures by shuttling between sun and shade, selecting colder microhabitats than those previously used, and adjusting activity periods (Kearney et al. 2009; Buckley et al. 2015; Gunderson and Leal 2015). Obviously, lizards must be careful thermoregulators in order to be able to behaviorally compensate for environmental warming, and the habitat must be thermally heterogeneous enough to allow selection of a significant variety of thermal microhabitats (Huey and Tewksbury 2009; Kearney et al. 2009; Goller et al. 2014; Scheffers et al. 2014; Sears and Angilletta 2015).

We studied the thermal biology of the Carpetan rock lizard, *Iberolacerta cyreni*, an accurate thermoregulator and cold‐specialist lizard (Monasterio et al. 2009; Aguado and Braña 2014) in the upper part of its distribution range. To assess the effect of global warming on thermal biology of lizards, we compared our own thermal information of *I. cyreni* from 25 years ago with present‐day data. In addition, we studied the thermal biology of *I. cyreni* under current climatic conditions using the protocol of Hertz et al. (1993). Thus, we obtained the preferred temperature range, the accuracy of thermoregulation, the thermal quality of the habitat, and the effectiveness of thermoregulation of *I. cyreni* under current climatic conditions. Finally, to quantify the actual rise of temperatures in a 25‐year period and to test the hypothesis of behavioral buffering of environmental warming in *I. cyreni*, we compared the available data from 25 years ago with present‐day information in the same area. As *I. cyreni* has the ability to accurately regulate its body temperature, we predict that lizards would be able to compensate somehow the impact of global warming in their body temperatures by means of their thermoregulatory behavior. We aimed to answer three main questions regarding the impact of climate change in Carpetan rock lizards: (1) Have ambient temperatures significantly increased in the habitat of *I. cyreni*?, (2) Has climate warming led lizards to a significant increase in body temperature?, and (3) Are lizards buffering the rise of environmental temperatures through their thermoregulatory behavior?

## Materials and Methods

### Species under study

**Figure 1:**
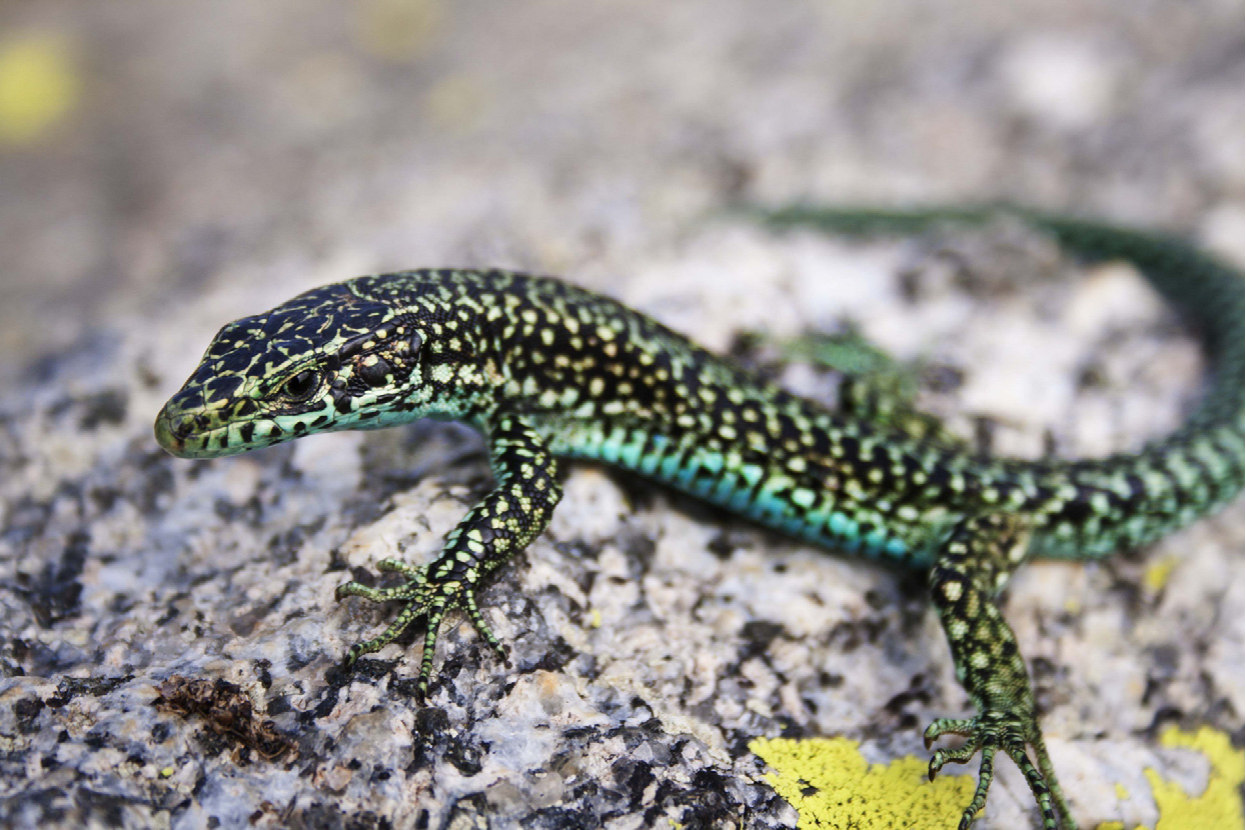
Male of *Iberolacerta cyreni* from the mountain chain of Sierra de Gredos (Spain).

The Carpetan rock lizard, *I. cyreni,* is a montane lizard endemic to central Spain that lives in isolated populations between 1600 and 2500 m (Pérez‐Mellado 1998; Fig. 1). *I. cyreni* is active between March and October (Pérez‐Mellado 1982, 1998) and prefers rocky and mixed shrub habitats (Martín and Salvador 1997b; Monasterio et al. 2010a,b). Carpetan rock lizards show a bimodal activity in summer, with a higher activity during mornings (Martín and Salvador 1997a; Aragón et al. 2001). This lizard species gains heat through heliothermy and thigmothermy and behaviorally regulates its body temperature mainly shuttling between microhabitats in sun and shade (Pérez‐Mellado 1982; Carrascal et al. 1992). Carpetan rock lizards are cold‐adapted reptiles and are able to change their thermal behavior under laboratory conditions to achieve body temperatures within their preferred range (Aguado and Braña 2014).

### Field temperatures

We sampled thermal biology of *I. cyreni* in the western part of the mountain chain of Sierra de Gredos (Spain; Fig. S2), at 2200 m, during summer from 1983 to 1989, as well as the summer of 2012, during the first 2 weeks of August. We captured active adult lizards by noosing during their daily activity period, from 09:00 to 18:00 GMT. Mean SVL of sampled lizards is 70.23 ± 0.42 mm (mean ± SE; *n* = 122), and mean weight is 7.34 ± 0.19 g (*n* = 118). Immediately after capture, we measured body temperature (*T*_b_), as well as air temperature (*T*_a_) 1 cm above the capture point, and substrate temperature (*T*_s_) of the capture point.

As a null hypothesis for thermoregulation, we measured operative temperature (*T*_e_) in August of 2012, simultaneously to *T*_b_ and in the same area. We employed nine cylindrical and unpainted copper models as null *T*_e_ models (Bakken and Angilletta 2014). We placed one thermocouple probe into each hollow model and connected it to a data logger HOBO H8 (^®^Onset Computer Corporation 470 MacArthur Blvd. Bourne, MA 02532) programmed to take a temperature record every 5 min, during 10 days. We randomly placed copper models in different microhabitats, obtaining 8115 measures of *T*_e_. Given the preference of rock lizards for rocky substrates (Martín and Salvador 1997b), and because the habitat is mainly made of big blocks of rock, we also measured different orientations of rocks as a main thermal microhabitat for thermoregulation. Thus, we obtained *T*_e_ for the following nine microhabitats: *flat rock*,* moss*,* soil*,* grass*,* rock facing south*,* rock facing east*,* rock facing north*,* rock facing west*, and *under rock*.

### Preferred temperature range

We measured selected body temperatures of *I. cyreni* during August of 2012 in a laboratory thermal gradient. We captured lizards from the same location of field sampling and immediately transported them to the laboratory. We provided water *ad libitum* to lizards during the experiment. In addition, we fed lizards with mealworms and crickets and housed them on individual terraria. We built the thermal gradient in a glass terrarium (100 × 60 × 60 cm) with a 150‐W infrared lamp over one of the sides, obtaining a gradient between 20°C and 60°C. We measured a selected temperature of a specific lizard each hour from 09:00 to 18:00 GMT with a digital thermometer. We used 24 adult lizards (12 males and 12 females), obtaining 156 selected temperatures. We considered the 50% of the central values of selected body temperatures as the preferred temperature range (PTR) to assess thermoregulation (Hertz et al. 1993; Blouin‐Demers and Nadeau 2005). We released lizards at the site of capture the next day after the experiment.

### Data analysis

To test the null hypothesis of thermoregulation and following the protocol developed by Hertz et al. (1993), we calculated accuracy of thermoregulation (mean *d*_b_), thermal quality of habitat (mean *d*_e_), and effectiveness of thermoregulation (mean *E*). We obtained effectiveness of thermoregulation by performing a bootstrap of 500 resamples, building pseudo‐distributions of three kinds of output values: accuracy (mean *d*_b_), thermal quality of the habitat (mean *d*_e_), and effectiveness of thermoregulation (mean *E*). We reported the values of arithmetic means of temperatures and indexes of thermoregulation with standard errors.

We performed parametric statistics when data followed the assumptions of normality and variance homogeneity. When data did not fulfill these assumptions, even after log transformations, we carried out nonparametric equivalent tests (Sokal and Rohlf 1995; Crawley 2007). We conducted all analyses on R, version 3.1.3 (R Core Team 2015), and we computed post hoc comparisons of Kruskal–Wallis tests with Nemenyi test with the package PMCMR (Pohlert 2014).

## Results

### Thermoregulation under present‐day climatic conditions

**Figure 2:**
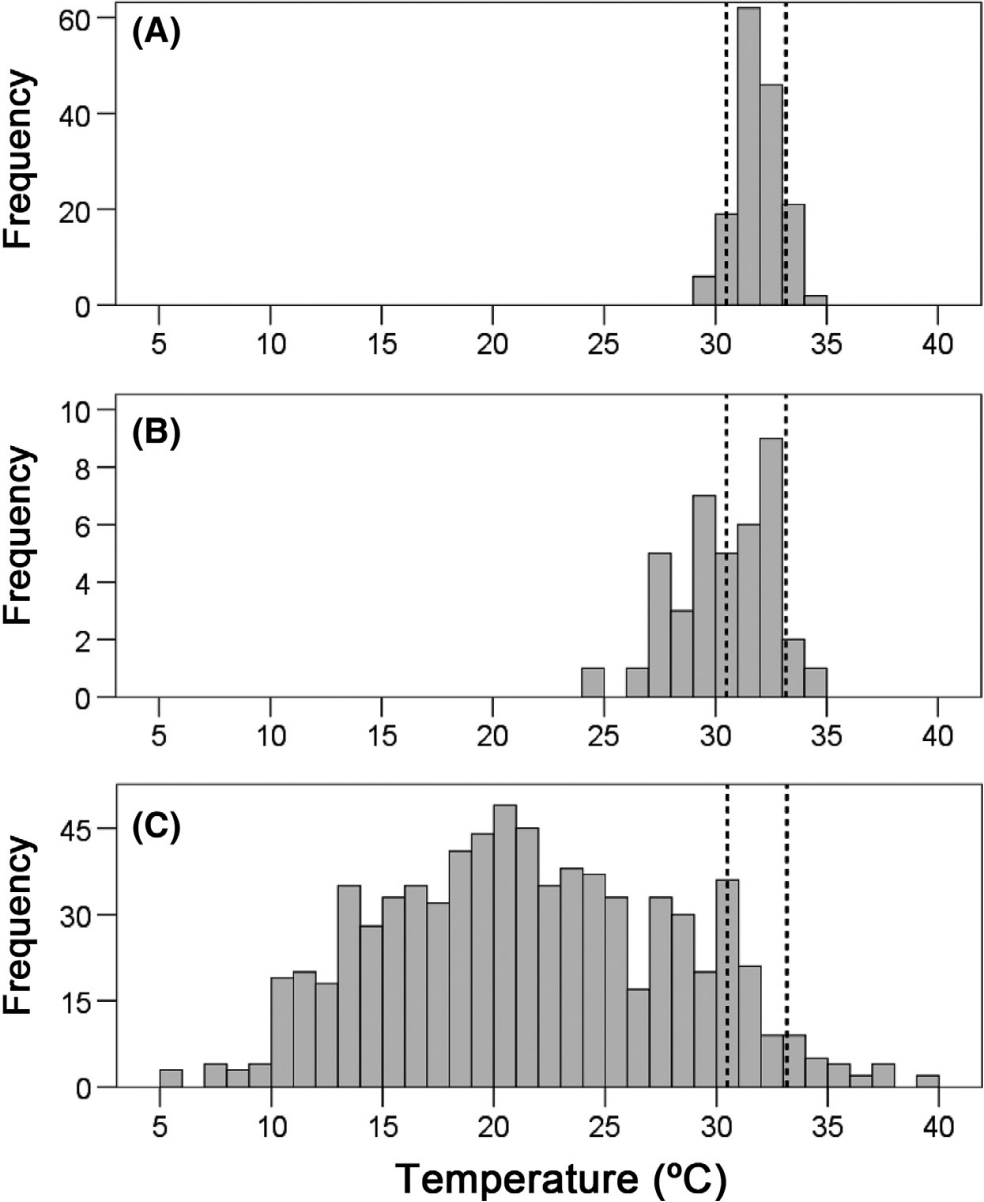
Temperatures of *Iberolacerta cyreni* of 2012: (A) selected body temperatures measured in thermal gradient to obtain the preferred temperature range (PTR), (B) field body temperatures of active lizards, and (C) operative temperatures as a null hypothesis of thermoregulation. Dotted lines delimit the PTR of *I. cyreni* (31.18–32.5°C).

The PTR of *I. cyreni* in the thermal gradient ranged from 31.18°C to 32.50°C (Fig. 2A). Thus, precision of thermoregulation of *I. cyreni* in summer was 1.32°C. For the sample of 2012, there were also not significant differences in body temperature (*T*_b_) by sex (males: 30.89 ± 0.42°C, *n* = 22; females: 29.65 ± 0.52°C, *n* = 18; ANOVA, *F*_1, 38_ = 3.542, *P* = 0.067). *T*_b_ of lizards reached the PTR from 10:00 to 11:00 GMT and from 13:00 to 15:00 GMT. Air temperatures (*T*_a_) were more than 3°C below the PTR during all day, and substrate temperatures (*T*_s_) only reached the PTR from 13:00 to 14:00 GMT and from 16:00 to 17:00 GMT.

Regarding the null hypothesis of thermoregulation, mean operative temperature (*T*_e_) was 21.60 ± 6.71°C (*n* = 8114). *T*_e_ of the studied microhabitats were significantly different (Kruskal–Wallis test, *H* = 1767.16, df = 8, *P* < 0.0001; Fig. S2). *Flat rock*,* moss,* and *rock facing south* showed similar distributions of *T*_e_ (*P* > 0.05 for Nemenyi paired comparisons in all cases) and were closest to the PTR among all microhabitats. On the other hand, *rock facing north*,* soil*, and *under rock* were the less suitable microhabitats, with similar distributions of *T*_e_ between them (*P* > 0.05 for paired comparisons in all cases). Finally, there were significant differences between *T*_e_ showed by *grass*,* rock facing east,* and *rock facing west* (*P* < 0.0001 in all cases). *T*_e_ of different thermal microhabitats changed along the daily activity period of lizards, so there was always a suitable (i.e., *T*_e_ within the PTR) place for thermoregulation (Fig. S2).

Finally, accuracy of thermoregulation (mean *d*_b_) of Carpetan rock lizards was 1.44 ± 0.025°C, thermal quality of habitat (mean *d*_e_) was 9.96 ± 0.024°C, and mean effectiveness of thermoregulation of rock lizards was 0.85 ± 0.003 (*n* = 40) for this population of *I. cyreni* under present‐day climatic conditions (Fig. 2).

### Comparing thermal biology after 25 years

**Figure 3:**
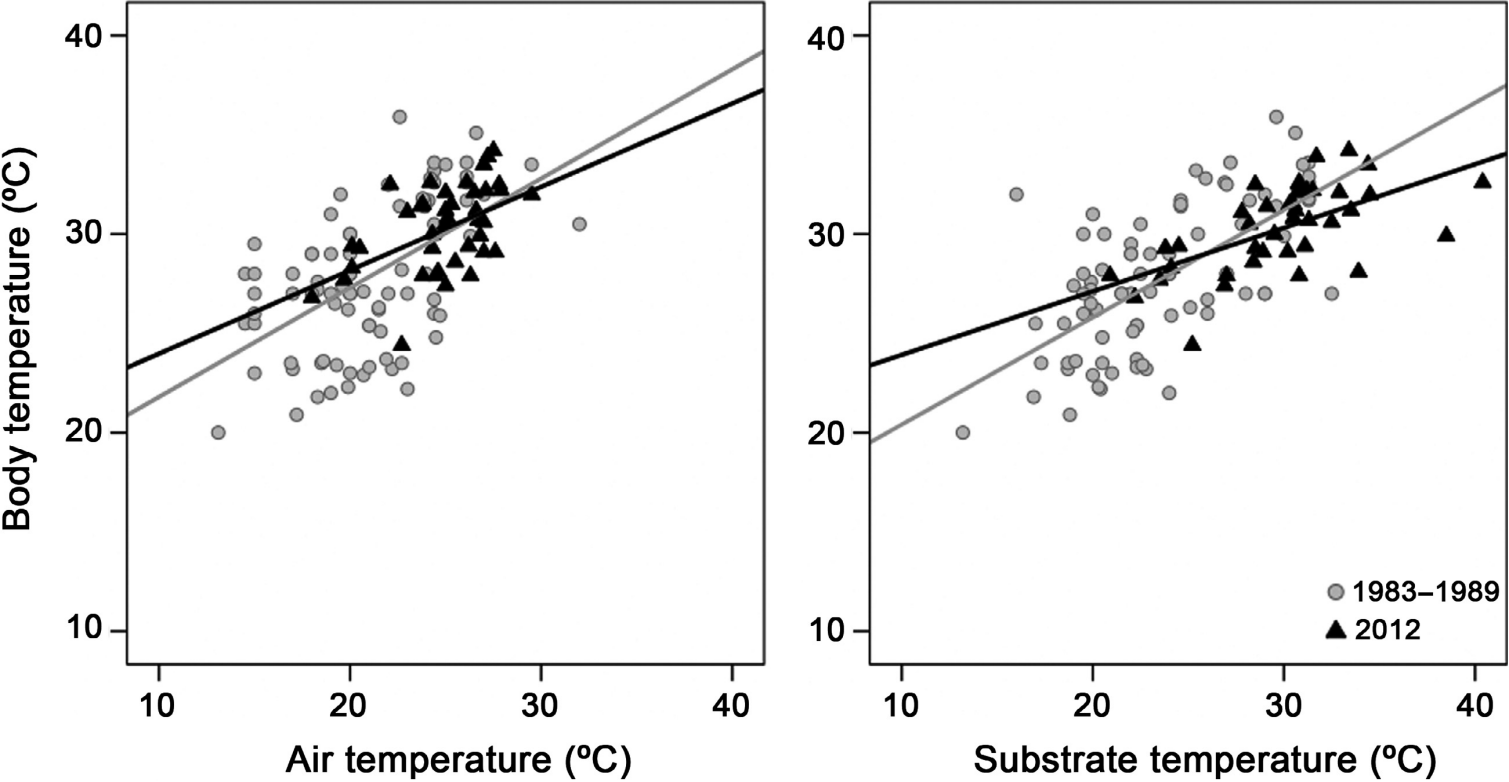
Simple linear regressions of air temperatures on body and substrate temperatures of *Iberolacerta cyreni* in the 1980s and under present‐day climatic conditions.

For the sample of 1983–1989, the body temperatures (*T*_b_) of males and females were similar (males: 28.02 ± 0.57°C, *n* = 39; females: 27.79 ± 0.56°C, *n* = 47; ANOVA, *F*_1, 84_ = 0.080, *P* = 0.778), so they were pooled together for further analyses. Body temperatures (*T*_b_) of *I. cyreni* significantly increased during the last 25 years (Mann–Whitney *U*‐test, *U* = 1009, *P* < 0.0001; Table 1). In addition, substrate temperatures (Mann–Whitney *U*‐test, *U* = 617, *P* < 0.0001), as well as *T*_a_ (ANOVA, *F*_1, 121_ = 45.591, *P* < 0.0001; Table 1), also increased. A two‐way ANOVA of temperature with the type of temperature (*T*_b_, *T*_a_, and *T*_s_) and the period (1983–1989 vs. 2012) resulted in a significant interaction between both factors (two‐way ANOVA, interaction: *F*_2_ = 6.128, *P* = 0.002). Thus, the increase in temperature between the 1980s and 2012 was different for *T*_b_, *T*_a_, and *T*_s_. The overall temperature change was significant although only moderate for *T*_b_, higher for *T*_a_, and even higher for *T*_s_. Furthermore, linear regression slopes remained unchanged from the 1980s to 2012, both for the relationship between *T*_b_ and *T*_a_ (sample of 1983–1989: *T*_b_ = 16.30 + 0.55 × *T*_a_, *R* ^2^ = 0.301; sample of 2012: *T*_b_ = 19.78 + 0.42 × *T*_a_, *R* ^2^ = 0.255; interaction term of ANCOVA, *F*_1_ = 0.411, *P* = 0.523; Fig. 3) as well as for the relationship between *T*_b_ and *T*_s_ (sample of 1983–1989: *T*_b_ = 15.01 + 0.54 × *T*_a_, *R* ^2^ = 0.415; sample of 2012: *T*_b_ = 20.72 + 0.32 × *T*_a_, *R* ^2^ = 0.366; interaction term of ANCOVA, *F*_1_ = 3.480, *P* = 0.065; Fig. 3).

**Table 1:** Mean temperatures of *Iberolacerta cyreni* for the sample of the 1980s and the present day sample in the same area of study. Mean temperatures ± SE (*N*)

	Sample of 1983–1989	Sample of 2012
Body temperature (*T*_b_)	27.87 ± 0.39 (87)	30.34 ± 0.34 (40)
Air temperature (*T*_a_)	21.13 ± 0.40 (84)	24.90 ± 0.40 (40)
Substrate temperature (*T*_s_)	23.77 ± 0.54 (83)	29.82 ± 0.64 (40)

## Discussion

Initially, the species of the genus *Iberolacerta* were largely distributed in the lower altitudes when the weather was colder. However, probably due to the increase in environmental temperatures and drought during the Messinian crisis, more than 5 Ma, they migrated to higher elevations (Arribas and Carranza 2004; Carranza et al. 2004; Crochet et al. 2004). Hence, *Iberolacerta* lizards have persisted in high mountain areas, where *I. cyreni* is been living since at least 1.5 Ma (Carranza et al. 2004). As a result, the high mountain areas offered a colder climate for lizards that had been predisposed to these conditions from millions of years ago, and thus the *Iberolacerta* lizard survived as a glacial relict.

Our results show a significant increase of more than 3°C in the environmental temperatures of the habitat of *I. cyreni* over approximately 25 years. Available data from a nearby weather station in La Covatilla (Salamanca, at 1960 m) reveal an increase in August temperatures from 2002 to 2011 of 2.5°C (≈0.28°C/year) for the monthly average maximum temperature, 3.4°C (≈0.38°C/year) for the monthly average minimum temperature, and 3.0°C (≈0.33°C/year) for mean monthly average mean temperature (data provided by the Spanish Meteorological Agency, “Agencia Española de Meteorología, AEMET”; available at http://www.aemet.es by request). Martín and Lopez (2013) reported an average warming of maximum temperature in June of 0.1°C/year, from data of a weather station at 1894 m, in the eastern area of the distributional range of *I. cyreni* (Puerto de Navacerrada). Thus, the increase in environmental temperatures in the area of study is consistently proven by our own results and substantiated with other available sources.

Within this scenario of a continued increase in temperature due to climate change, lizards would have two possibilities to avoid extinction: to migrate to new thermal suitable habitats or to adapt to new thermal conditions of their habitats (Berg et al. 2010; Chevin et al. 2010; Gunderson and Stillman 2015). For montane lizards, especially those living in mountain chains with an east–west orientation, as is the case for *Iberolacerta* of Central System at the Iberian Peninsula, it is virtually impossible to migrate to colder habitats (Araújo et al. 2006; Huey et al. 2012), because populations are isolated in several areas separated by lowlands and valleys replete with unsuitable thermal conditions. The lower limits of the distribution of *I. cyreni* (1600 m als) are a barrier to their expansion (Monasterio et al. 2011). Hence, montane lizards would need to adapt themselves to warmer conditions of their habitats (Berg et al. 2010; Chevin et al. 2010). To do this, lizards would have two options: (1) plasticity or evolution of thermal physiology, or (2) buffering of warming through behavioral thermoregulation (Huey and Tewksbury 2009; Kearney et al. 2009). The extent of phenotypic plasticity of thermal traits appears to be limited, especially for the critical temperature maximum, which is what cold‐adapted lizards would need to increase in order to allow preservation of the species under global warming (Gunderson and Stillman 2015). Therefore, high mountain lizards would have to resort to the flexibility of their thermoregulatory behavior to survive in a habitat that is under continuous warming (Huey et al. 2012; Gunderson and Stillman 2015). In addition, the ability to buffer the impact of the warming of the habitat might be more important for thermal specialists than for thermal generalist ectotherms (Huey et al. 2012). This is because the thermal fitness curves are left‐skewed: fitness increases gradually from the critical thermal minimum to the optimal temperature and it sharply declines once body temperatures exceed the optimal temperature (Martin and Huey 2008; Angilletta et al. 2010). Due to this asymmetry, a decrease in body temperature entails a smaller decrease in fitness than a similar increase in body temperature, due to a mathematical property known as Jensen's inequality (Martin and Huey 2008; Huey et al. 2012). Furthermore, the negative effect of exceeding the optimum temperature on fitness is greater as the thermal curve is narrower, so the more thermal specialist the population, the more vulnerable it would be to global warming (Huey et al. 2012).

The PTR of lizards approximately fits the thermal optimal for fitness, although it may be somewhat lower (Hertz et al. 1993; Martin and Huey 2008). *I. cyreni* shows a PTR of a cold‐adapted specialist, ranging from 31.18°C to 32.50°C, with a breadth of only 1.32°C. In fact, the PTR of *I. cyreni* is one of the narrowest and coldest among the Lacertidae (Bauwens et al. 1995; Aguado and Braña 2014). Our results on thermal preferences are similar to those obtained in previous studies of the same species (Martín and Salvador 1993; Bauwens et al. 1995). Nonetheless, the range reported here is somewhat lower than the range found by Aguado and Braña (2014). These differences could be due to the effects of seasonality, as it is common in other species (see, for example, Díaz et al. 2006). The fact that the PTR is lower than the physiological optimal temperature shows that “suboptimal is optimal” for *I. cyreni* lizards (Martin and Huey 2008; Fig 1). Further evidence of the adaptation of *I. cyreni* to cold temperatures is that lizards thermoregulate more carefully as populations live at higher altitudes. Monasterio et al. (2009) reported an effectiveness of thermoregulation of the Carpetan rock lizard of 0.52 at 1700 m and 0.70 at 1900 m of altitude in summer, and Aguado and Braña (2014) reported an effectiveness of thermoregulation of 0.78 for *I. cyreni* at 1800–2000 m in spring. Here, we report an effectiveness of thermoregulation of 0.85 at 2200 m of altitude in summer. This positive relation between effectiveness of thermoregulation and elevation appears to be due to the trend for worst thermal quality of habitat with altitude, given our results and those from Monasterio et al. (2009). These findings provide evidence that lizards are able to maintain their body temperatures relatively closer to the optimum while the habitat is becoming colder at increasing altitudes. In short, *I. cyreni* is a cold‐adapted thermal specialist, like the other studied species of *Iberolacerta* (Martín and Salvador 1993; Aguado and Braña 2014; Ortega et al. 2016; Žagar et al. 2015).

The effective thermoregulation of *I. cyreni* lizards makes them good candidates to behaviorally buffer the impact of global warming (Huey and Tewksbury 2009; Kearney et al. 2009). Furthermore, it has been recently demonstrated that *I. cyreni* lizards are able to modify their behavior in order to thermoregulate effectively under different ambient conditions in the laboratory (Aguado and Braña 2014). While air temperatures of the habitat of *I. cyreni* have increased more than 3.5°C, and substrate temperatures by 6°C, from the 1980s to 2012, body temperatures of lizards have increased less than 2°C. Therefore, Carpetan rock lizards are buffering the impact of global warming. The linear relationship between body and air temperature and between body and substrate temperature remains similar as it was 25 years ago. This result would discard the possibility that buffering of global warming could be achieved by changing heating rates of lizards, or a similar physiological adaptation. Therefore, the extent to which body temperature is lower than it should be in proportion to the warming of environmental temperatures could be attributable to the thermoregulatory behavior. In other words, our results suggest that *I. cyreni* lizards have been taking advantage of their thermoregulatory behavior within the last 25 years of global warming, probably selecting increasingly colder microhabitats in proportion to their availability in the habitat.

**Figure 4:**
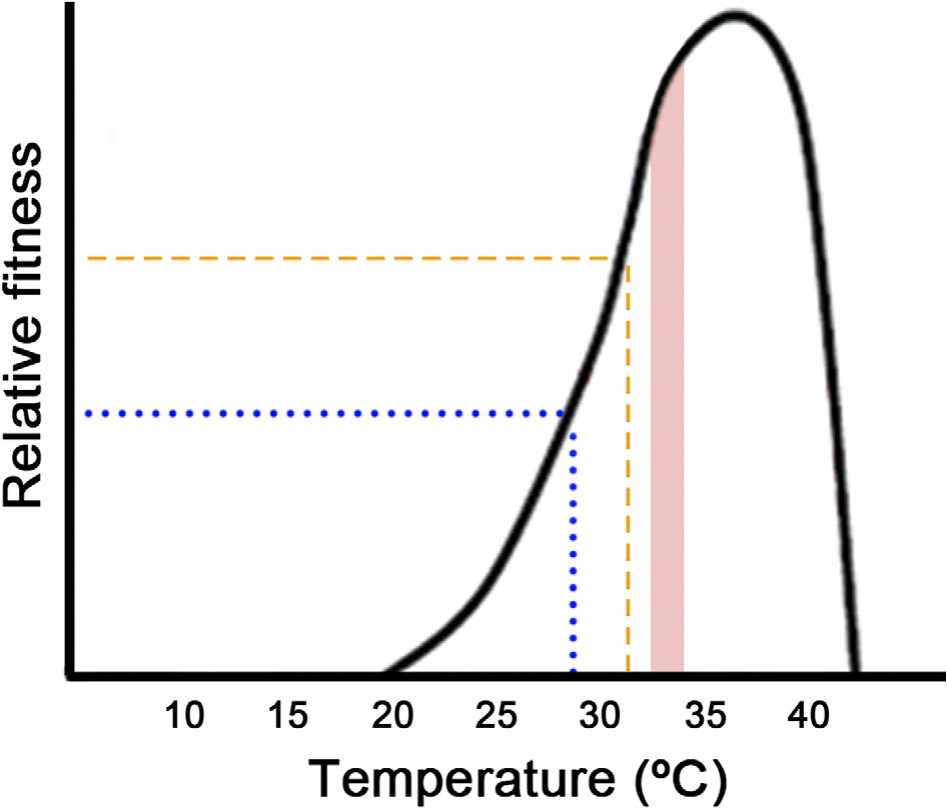
Theoretical curve of thermal fitness in *Iberolacerta cyreni* (according to data of running performance reported by Bauwens et al. 1995). The shaded area is the preferred temperature range of *I. cyreni* in summer of 2012 (present study). The dotted line is the mean body temperature from 25 years ago, and the dashed line is the mean body temperature of present‐day lizards. These lines are represented with correspondingly approximate fitness as diagram of figure 1 of Huey et al. (2012), to illustrate the potential situation of *I. cyreni* given available data.

Huey et al. (2012) emphasize that a key point to vulnerability of a species to global warming is how colder both operative and field body temperatures were in relation to the optimal temperature at the beginning of global warming. If body temperatures were colder than the optimal temperature, then warming will enhance fitness until body temperatures would exceed optimal temperature (Huey et al. 2012). We lack data of body temperatures of *I. cyreni* at the beginning of global warming. However, we report in this study that mean body temperatures during summer from 25 years ago were more than 6°C colder than the optimal temperature of physiological performance, which is an estimate of the optimal temperature for fitness (34.53 ± 0.50°C; Bauwens et al. 1995). In addition, present‐day summer body temperatures of *I. cyreni* are more than 4°C colder than optimal fitness temperatures. Therefore, our data support that the fitness of *I. cyreni* might have even increased for this period of 25 years of climate change, and there is a margin of safety for a potential rise of approximately 5°C in body temperatures until fitness would decline (see Fig. 4, based in Bauwens et al. 1995 and Huey et al. 2012). However, we must remember that the detrimental effect of the rapid increase in environmental temperatures may be stronger for eggs and hatchlings of *I. cyreni*, and, probably, other *Iberolacerta*, lizards (Monasterio et al. 2016).

In conclusion, the habitat of *I. cyreni* has warmed significantly in the past 25 years, and more warming and drought is predicted during this century (Nogués‐Bravo et al. 2008). Carpetan rock lizards are cold‐adapted specialists, a condition that increases their vulnerability to global warming. Evolutionary history of the *Iberolacerta* indicates that their strategy was to retreat to colder areas during climate warming of past ages (Carranza et al. 2004; Crochet et al. 2004). However, lizards no longer have a place to move in the face of anthropogenic global warming (Araújo et al. 2006). Hence, behavioral compensation seems to be the sole mechanism for montane lizards to avoid extinction. Furthermore, the reported increase in body temperatures of Carpetan rock lizards might have improved fitness. Chamaillé‐Jammes et al. (2006) found a positive effect in the fitness of *Zootoca vivipara* for a studied period of 18 years of global warming, so it is possible that the same would happen to *I. cyreni*. In addition, current body temperatures are still colder than the optimal temperature of lizards. Consequently, lizards still have a wide safety margin until fitness would decline. Nonetheless, it is important to keep in mind that once behavioral buffering would be too costly and body temperatures would pass the optimal temperature, the large decline of fitness of such thermal specialists due to a slight increase in body temperature, along with the inability to migrate, would lead montane lizards to a dead end of extinction.

## Conflict of Interest

None declared.

## Supporting information


**Figure S1.** Study area in the western part of the mountain chain Sierra de Gredos (Spain).

**Figure S2.** Operative temperatures of different thermal microhabitats are provided for each hour of the day (GMT) for the sample of 2012. Dotted lines provide the preferred temperature range of *Iberolacerta cyreni* (31.18–32.5°C).
