# Supplementary figures and images for: Behavioral buffering of global warming in a cold‐adapted lizard
Source: Ecol Evol. 2016 Jun 12;6(13):4582–90. doi: 10.1002/ece3.2216 (PMC4931003; doi:10.1002/ece3.2216)

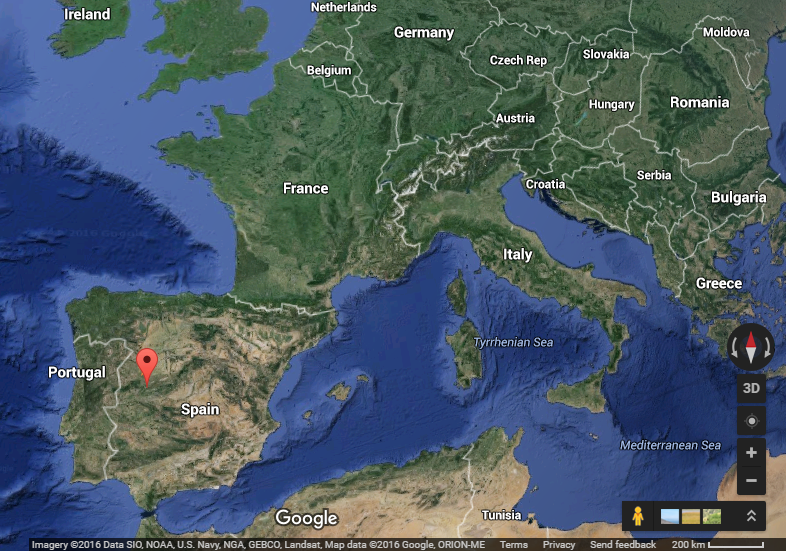

Supplement: Supplementary file 1 — Figure S1. Study area in the western part of the mountain chain Sierra de Gredos (Spain). [file ECE3-6-4582-s001.tif]

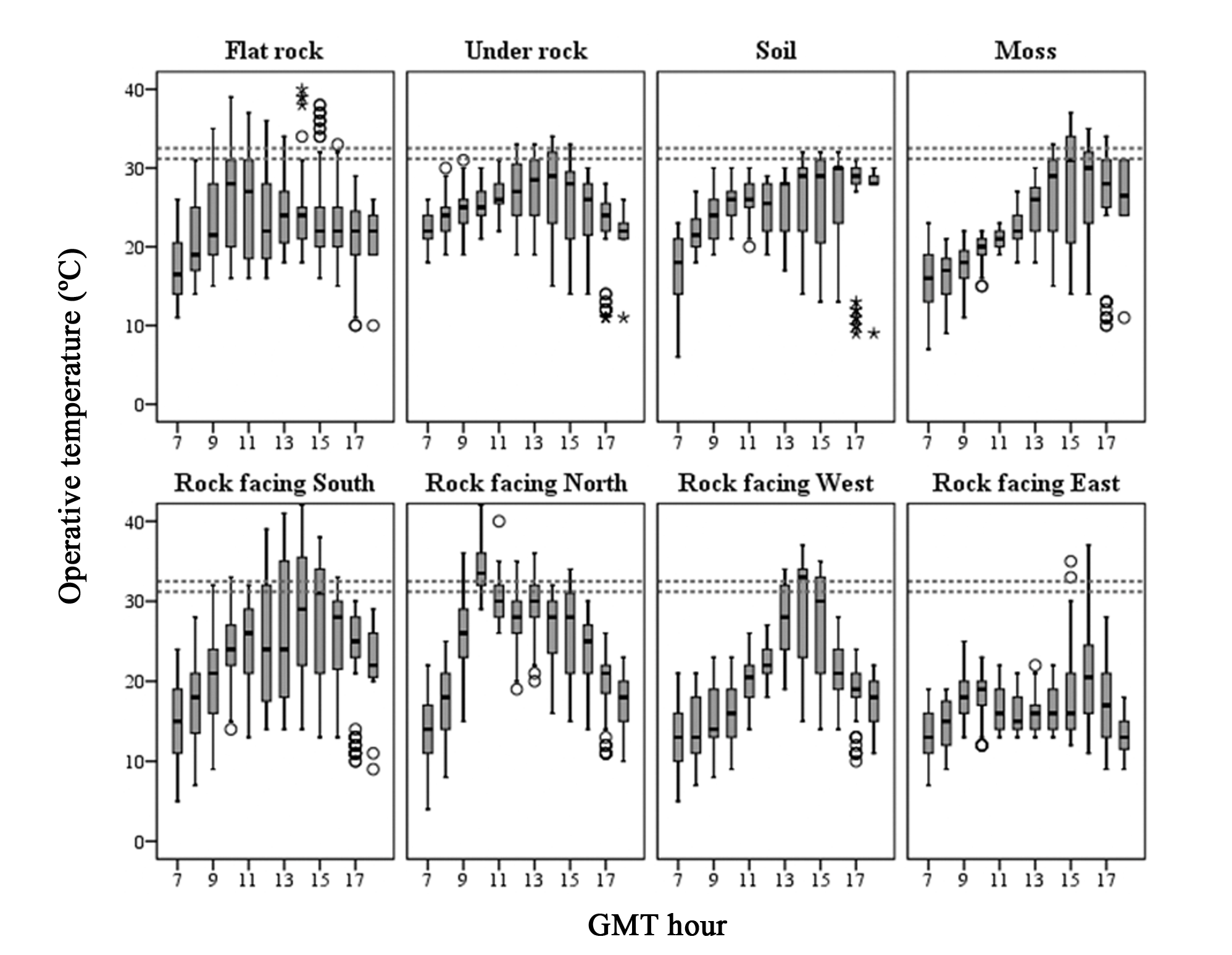

Supplement: Supplementary file 2 — Figure S2. Operative temperatures of different thermal microhabitats are provided for each hour of the day (GMT) for the sample of 2012. Dotted lines provide the preferred temperature range of Iberolacerta cyreni (31.18–32.5°C). [file ECE3-6-4582-s002.tif]
